# Prevalence of *Schistosoma mansoni* infection in Ethiopia: a systematic review and meta-analysis

**DOI:** 10.1186/s40794-020-00127-x

**Published:** 2021-02-01

**Authors:** Siraj Hussen, Demissie Assegu, Birkneh Tilahun Tadesse, Techalew Shimelis

**Affiliations:** 1 School of Medical Laboratory Science, College of Medicine and Health Sciences, Hawassa University, Hawassa, Ethiopia; 2 School of Medicine, College of Medicine and Health Sciences, Hawassa University, Hawassa, Ethiopia

**Keywords:** Meta-analysis, *S. mansoni*, Prevalence, Ethiopia

## Abstract

**Background:**

Schistosomiasis is a common helminthic infection in the tropics and subtropics, particularly in sub-Saharan African countries including Ethiopia. In these counties, *Schistosoma mansoni* infection is a significant public health problem due to the risk of reinfection and recurrent disease despite implementing several rounds preventive chemotherapy. This systematic review and meta-analysis aimed at assessing the pooled prevalence of schistosomiasis in Ethiopia.

**Methods:**

The PRISMA guidelines were followed to perform the systematic review and meta-analysis. Published studies from January 1999 to June 2020 were searched in Medline, PubMed, Google Scholar, EMBASE, HINARI, and Cochrane Library using key words including: “prevalence”, “incidence”, “schistosomiasis” “Bilharziasis”, “Bilharzia”, “*S. mansoni* “, “Ethiopia”. Heterogeneity of included studies was assessed using Cochran’s Q test and *I*^*2*^ test statistics while publication bias was assessed using Egger’s test.

**Results:**

Ninety-four studies were included in the systematic review and meta-analysis. The pooled prevalence of *S. mansoni* in Ethiopia was 18.0% (95%CI: 14.0–23.0). The southern region of Ethiopia had a higher *S. mansoni* prevalence of 25.9% (995% CI, 14.9–41.1) than the national prevalence. The burden of *S. mansoni* infection was also higher than the national average in rural areas and among men with pooled prevalence of 20.2% (95% CI, 13.2–28.5) and 28.5% (95%CI, 22.7,35.1), respectively. The trend analysis showed that the prevalence of *S. mansoni* infection in Ethiopia decreased over the past 15 years, potentially because of the repeated preventive chemotherapy.

**Conclusion:**

The review unveiled a moderate prevalence of *S. mansoni* infection in Ethiopia. Targeted treatment of at-risk population groups ad high burden areas coupled with implementation of integrated vector control strategies are critical to address the burden of Schistosomiasis.

**Supplementary Information:**

The online version contains supplementary material available at 10.1186/s40794-020-00127-x.

## Background

Schistosomiasis is the most widely distributed chronic but neglected tropical disease (NTD) that affects people living in communities where there is poor environmental sanitation and water supply [1]. Schistosomiasis is one of the deadliest NTDs mostly caused by three different species of blood-dwelling fluke worms of the genus *Schistosoma*, namely, *S. haematobium*, which causes urinary schistosomiasis, and *S. mansoni* and *S. japonicum*, which cause intestinal schistosomiasis. Human schistosomiasis is second only to malaria in mortality [1, 2]. An estimated 700 million people in 76 countries are at risk of schistosomiasis, and 240 million people are infected. About 85% of the infections occur in Africa with an estimated annual death of 280,000 people [2–4].

There is evidence to suggest that *S. mansoni* could interact with other chronic infections like HIV [5]. It has been shown that treating *S. mansoni* infections reduces the risk of HIV transmission in female adults. Treatment with praziquantel remains the mainstay of therapy in the absence of approved safe and effective vaccines. Mass drug administration (MDA) has been practiced by many countries as the sole intervention to control schistosomiasis; however, there is a need to closely monitor these interventions and understand their effect to the host and the schistosomes [6].

In addition to the high mortality, *S. mansoni* infection in school-age children, adolescents and young adults is associated with growth delay, anemia and vitamin-A deficiency as well as possible cognitive and memory impairment limiting their potentials in learning [7]. Signs and symptoms of schistosomiasis vary based on the intensity of infection and location of species-specific egg trapped in the tissues [3]. In sub-Saharan African countries including Ethiopia, *S. mansoni* is the main cause of clinical abnormalities such as hepatomegaly, splenomegaly, and periportal fibrosis [8].

Schistosomiasis is more widespread in poor rural communities particularly in places where fishing and agricultural activities are dominant. Domestic activities such as washing clothes and fetching water in infected waters expose women and children to infection. Poor hygiene and recreational activities like swimming and fishing also increase the risk of infection in children [9].

In Ethiopia, about 5.01 million people are infected with schistosomiasis and 37.5 million people are at an increased risk of infection [10]. *S. mansoni* infection is reported in all administrative regions and is rapidly spreading in connection with water resource development and intensive population movements [11]. On the other hand, other species of Schistosoma including *S. haematobium* are less prevalent in Ethiopia due to the high altitude. The optimal altitude category for high transmission of *S. mansoni* is between 1000 and 2000 m [12]. Two species of freshwater snails (*Biomphalaria pfeifferi* and *Biomphalaria sudanica)* are responsible for the transmission of this parasite in Ethiopia [13]. The distribution *of S. haematobium* is restricted to three lowland areas (Awash and Wabeshebele river basins and from Kurmuk at the Ethiopia/Sudan border), and snails, mainly *Bulinus abyssinicus* and *Bulinus africanus*, are intermediate hosts [14].

The national control program is designed to break transmission of neglected diseases and other poverty related infections by 2025. Estimates of *S. mansoni* prevalence vary widely and lack consistency across different regions of the county. There is a need for updated and summarized data on the extent of disease to facilitate effective prevention and control programs. In this review, we used data published from Ethiopia between 1999 and 2020 to perform a systematic review and meta-analysis of the prevalence of *S. mansoni*.

## Methods

### Search strategy

A comprehensive literature search of articles published between January 1999 and June 2020 was conducted from biomedical databases: Medline, PubMed, Google Scholar, EMBASE, HINARI, and Cochrane Library using a special index search terms (medical subject headings (MeSH): “prevalence”,“incidence”, “schistosomiasis”, “Bilharziasis”, “Bilharzia”, “Parasite ““*S. mansoni* “, “Ethiopia”, and each sub-region in Ethiopia. Search terms were combined with Boolean terms ‘AND’/‘OR’. Studies published in English langiage in humans were considered since in Ethiopia, the official language for scientific communication is English, and it is unlikely to find studies/survey results published in the local languages. Age groups were defined as 14 years or younger (children); 15–17 years (adolescents); and 18 years or older (adults). The Preferred Reporting Items for Systematic Reviews and Meta-analyses (PRISMA) guideline was used to report the result of this systematic review and meta-analyses (Additional file 1).

### Selection criteria

Abstracts retrieved from the initial search were screened using the inclusion and exclusion criteria as defined below.

### Inclusion criteria and exclusion criteria

Studies were selected for systematic review and meta-analysis if: 1) they were conducted in Ethiopia; 2) study design was cross-sectional; 3) studies reported the prevalence of *S. mansoni* in the stool specimens 4) studies reported data in humans and were published in the English language. Studies which analyzed urine antigen for method comparison were excluded. No age limitation was proffered. Studies which employed circulating-cathodic-antigen (CCA)/polymerase chain reaction (PCR) techniques for the diagnosis of schistosomiasis were excluded to reduce heterogeneity.

Studies were examined for eligibility using titles and abstracts. Relevant abstracts were further assessed for inclusion in the list of full text articles. During the article selection process, studies which did not have full texts were excluded since it was not possible to assess the quality of each article in the absence of their full texts.

### Data extraction

Titles and abstracts of all identified articles were screened by SH, TS, BTT and DA. For studies that met the predefined eligibility criteria, full-text articles were obtained and reviewed by SH and DA. Data were extracted independently and in duplicate for all studies by SH and DA using extraction tool built in Microsoft Excel 2013. Discrepancies between SH and DA were resolved by TS. The data abstraction format included first author, study design, region in Ethiopia, publication year, sample size, study population, number who tested positive and prevalence of *S. mansoni*.

### Quality assessment

The quality of each article was assessed using 9-point Joanna Briggs Institute (JBI) critical appraisal tools [15]. The tool uses the following criteria: 1) sample frame appropriate to address the target population, 2) study participants sampled in an appropriate way, 3) adequate sample size, 4) Study subjects and the setting described in detail, 5) the data analysis conducted with sufficient coverage of the identified sample, 6) valid methods were used for the identification of the condition, 7) the condition was measured in a standard and reliable way for all participants, 8) appropriate statistical analysis; and, 9) adequate response rate. Individual studies were assigned a score that was computed using different parameters in line with the review objectives. The responses were scored 0 for “Not reported” and 1 for “Yes”. Total scores ranged between 0 to 9. Studies with medium (fulfilling 50% of quality assessment parameter) and high quality were included for analysis [15]. None of the studies were excluded based on the quality assessment criteria (Additional file 2).

### Statistical analysis

Data entry and analysis was done using Comprehensive Meta-analysis (version 3.1, Biostat, Eaglewood, USA). The overall effect (pooled estimated effect size) of *S. mansoni* prevalence with 95% confidence interval (CI) was estimated using random-effects meta-analysis (random effects model) to account for heterogeneity of the included studies.

### Sub-group analysis

Sub-group analysis was performed based on geographical region (Amhara, Oromia, Southern Ethiopia, Tigray, Harari and Afar), Year of study (1999–2005, 2006–2010, 2011–2015 and 2016–2020), laboratory diagnostic test (Kato-Katz, Kato-Katz and wet mount, wet mount and formol-ether, formol-ether, Kato-Katz and formol-ether, Kato-Katz and Sodium acetate-acetic acid-formalin SAF and wet mount), age groups (all age groups, children, children and adolescents, adolescents and adults, and adults), sex (male and female), and study setting (rural and urban). Prevalence data were also estimated by Woreda, which are the second smallest administrative units in the country.

### Heterogeneity and publication bias

Statistical heterogeneity was assessed by Cochran’s Q test and *I*^*2*^ statistic. The *I*^*2*^ offers an estimate percentage of the variability in effect estimates that is due to heterogeneity rather than sampling error or chance differences. The presence of heterogeneity was confirmed using Cochran’s Q test with *p*-value < 0.10 showing statistically significant heterogeneity [16]. Furthermore, *I*^*2*^ test was used to measure level of statistical heterogeneity between studies (values of 25, 50 and 75% are low, medium, and high heterogeneity, respectively) [17]. Tau-square test was also used. Publication bias was assessed using the Egger weighted regression test (*P* < 0.05) [18].

## Results

### Identified studies

**Fig. 1:**
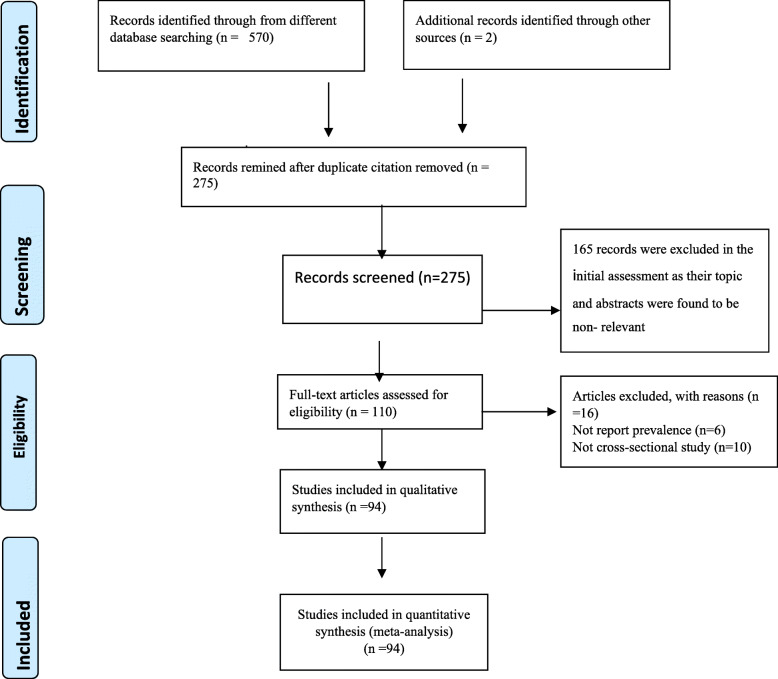
Flow diagram of the studies included in the Meta-analysis

A total of 570 records were retrieved through electronic database searching. A total of 165 articles were excluded in the initial assessment as their topic and abstracts were found to be non-relevant. Ninety-four articles were assessed for eligibility; 16 articles were excluded (ten articles were not cross-sectional study and six had no prevalence data). Finally, 94 studies were found to be eligible and were included in the meta–analysis (Fig. 1).

### Study characterstics

**Fig. 2:**
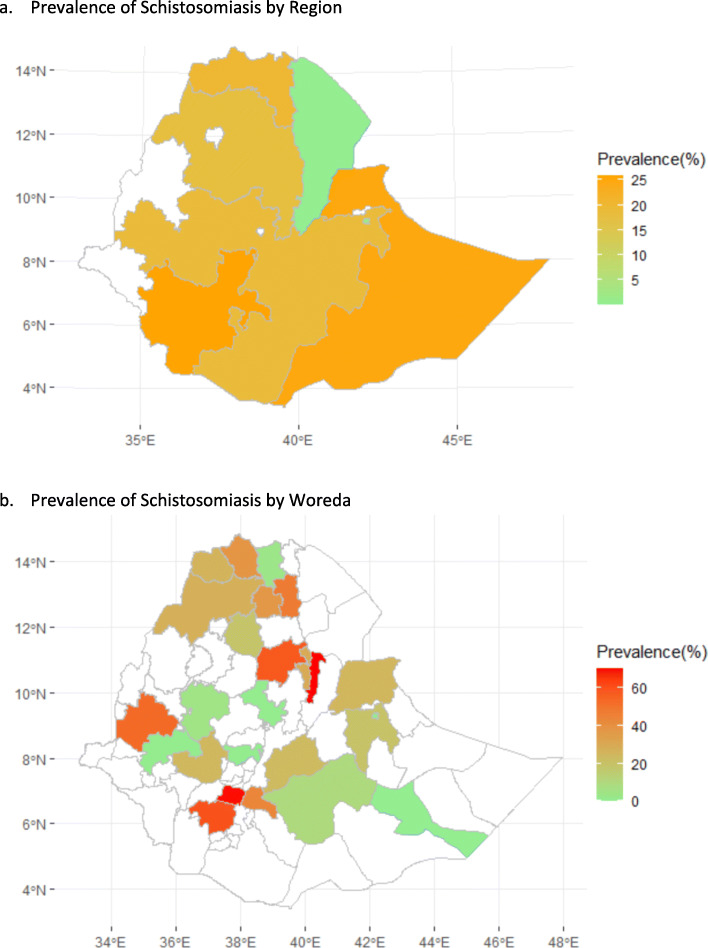
Prevalence of *S. mansoni* by Region and Woreda in Ethiopia. **a** Prevalence of Schistosomiasis by Region. **b** Prevalence of Schistosomiasis by Woreda*. *Woreda represents the second smallest adminstrative units in Ethiopia

**Fig. 3:**
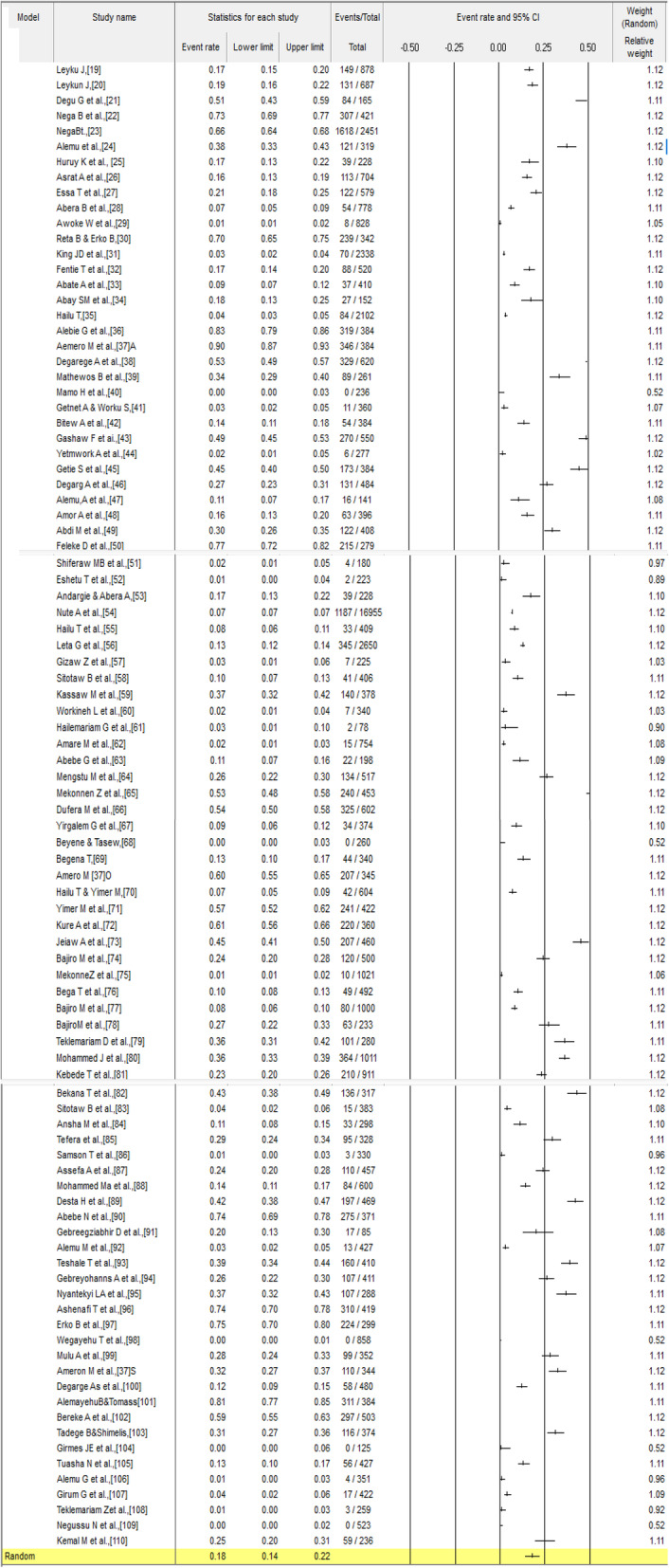
Forest plot for the prevalence of *S. mansoni* in Ethiopia

**Fig. 4:**
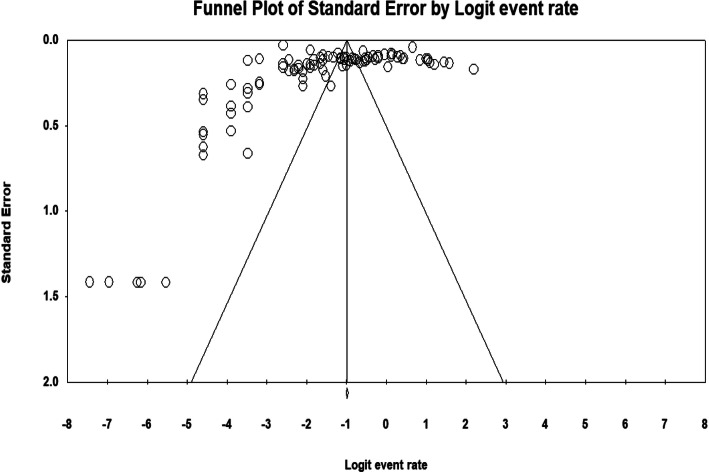
Funnel plot for the prevalence of *Schistosoma mansoni* in Ethiopia

A total of 64,189 participants with *S. mansoni* infectio were included in the review. Included studies were published between 1999 and 2020 and were reported from the seven administrative regions of Ethiopia: Amhara, Oromia, Southern Ethiopia, Tigray, Harar, Afar, and Somali (Additional file 3 and Fig. 2). The sample size of studies varied from 78 to 16,955 participants (Additional file 3). The highest and lowest prevalence of *S. mansoni* infection was reported in Amhara (89.6%) and Southern Ethiopia (0.12%), respectively (Additional file 3). The pooled prevalence of *S. mansoni* infection among Ethiopian population was 18% (95%CI: 14.0–22.0) (Fig. 3). As the test statistic showed significant heterogeneity among studies (I^2^ = 99.234, *p* < 0.001), and Tau-squared = 1.167, the Random effects model was used; however, no evidence of publication bias was shown with Egger’s regression intercept (*p* = 0.416) (Additional file 4). The symmetry of funnel plot shows a small publication bias and insignificant effect as portrayed graphically (Fig. 4).

### Subgroup analysis

Subgroup analysis revealed a broad inconsistency in the prevalence of *S. mansoni* infection within in groups (Table 1). Prevalence of *S. mansoni* infection was the highest in 2006 to 2010 publications at 28.0% (95% CI, 11.0–55.0), while the lowest prevalence was in studies published between 1999 and 2005 (9.9, 95% CI, 4.6–19.9). By region, the northern regions of the country had a higher *S. mansoni* infection burden as compared to the southern regions (Table 1). Prevalence was also higher in rural areas 20.2% (95% CI: 13.2–28.5) than urban areas 16.5% (95% CI: 11.2–23.7) (*p*-value < 0.001).

**Table 1:** Sub-group prevalence estimates for *S. mansoni* in Ethiopia

Study parameters	Subgroup analysis	Studies included	Sample size	No. pos	Pre.%(95% CI)	I^2^%	Q-p
Study year	1999–2005	6	2560	392	9.9(4.6,19.9)	97.280	***
	2006–2010	5	4112	2063	28.0 (11.0,55.0)	99.227	***
	2011–2015	48	24,261	6196	22.6 (16.7,29.7)	99.021	***
	2016–2020	35	33,256	4293	13.7 (9.8,18.7)	98.965	***
Geographical Region	Amhara	42	41,444	7213	17.5 (11.9,25.1)	99.490	***
	Oromia	26	12,541	2995	18.7 (13.4,25.5)	98.562	***
	Tgray	9	3560	966	20.2 (11.2,33.6)	98.425	***
	Southern	13	5204	1691	25.9 (14.9,41.1)	98.706	***
	Harar	2	681	20	2.3 (0.6,8.3)	77.505	**
	Afar	1	523	0	0.1 (0.0,1.5)	0.000	*
	Somali	1	236	59	25 (19.9,30.9)	0.000	*
Study setting	Rural& urban	27	11,339	2607	16.1 (10.8,23.2)	98.636	***
	Rural	41	41,456	8039	20.2 (13.2,28.5)	99.530	***
	Urban	26	11,394	2298	16.5 (11.2,23.7)	98.523	***
Age groups	All age group	51	29,093	7942	22.6 (17.1,29.2)	99.155	***
	Children	11	4004	859	13.7 (7.2,24.5)	98.365	***
	Children&adolescent	26	28,851	3747	15.7 (9.5,24.8)	99.395	***
	Adolescent&adult	5	2018	394	5.0 (1.1,20.4)	97.447	***
	Adult	1	223	2	1.0 (0.3,3.6)	0.00	***
Diagnostic method	Kato-Katz	43	22,865	8037	30.8 (24.6,37.8)	98.958	***
	Kato-Katz & wet mount	9	4198	675	14.9 (8.7,24.5)	97.819	***
	Wet mount & formol-ether	19	24,090	2131	6.8 (3.7,12.1)	98.941	***
	Formol-ether	8	3561	456	10.8 (6.1,18.5)	97.065	***
	Kato-Katz & formol-ether	7	2940	1099	32.3 (13.8,58.6)	99.177	***
	Kato-Katz &SAF	3	3096	419	19.8 (0.9,86.4)	99.750	***
	wet mount	5	3439	127	2.9 (1.6,5.2)	84.150	***
Sex	Male	50	22,684	7372	28.5 (22.7,35.1)	98.802	***
	Female	50	22,684	5947	21.2 (16.5,26.8)	98.671	***

*NB* CS=Cross-sectional, *NR* Not reported, *formol-ether* Formaline ether concentration technique, *SAF* Sodium acetate-acetic acid-formalin, *Yrs* Years, *NR* Not reported, *No. pos* Number positive, *Pre* Prevalence, *Q_S* Quality score, Cochran’s *p*-value. *** *p*-value < 0.001; **p-value< 0.05; *p-value > 0.05

We also compared the pooled prevalence of *S. mansoni* by diagnostic method, which showed significant variability – 30.8% (95%CI: 24.6–37.8) using Kato-Katz, 14.9% (95%CI: 8.7–24.5) using Kato-Katz & wet mount, 6.8% (95%CI: 3.7–12.1) using wet mount & formol-ether, 10.8% (95%CI: 6.1–18.5) using Formol-ether, 32.3% (95%CI: 13.8–58.6) using Kato-Katz & formol-ether, 19.8% (95%CI: 0.9–86.4) using Kato-Katz &SAF, and 2.9% (95%CI: 1.6–5.2) using wet mount (Table 1).

The burden of infection also varied by age and sex. Male participants had a higher burden of *S. mansoni* infection (28.5% (95%CI: 22.7–35.1) than female participants (21.2% (95%CI: 16.5–26.5). By age, infection burden was the highest in children/adolescents,15.7% (95% CI: 9.5–24.8), compared to that in adolescents/adults 5.0% (95% CI: 1.1–20.4) (Table 1).

We finally ran a meta-regression analysis to identify the independent predictors of burden of *S. mansoni* infection, which showed that geographic region (*p* = 0.031), and laboratory diagnostic technique (*p* < 0.001) indecently predicted *S. mansoni* infection (Additional file 5).

## Discussion

Efforts to reduce the epidemiological and clinical consequences of schistosomiasis through the deworming program of the Ethiopian Enhanced Outreach Strategy targeting children below five years of age has been in progress since 2010. Progress is mainly due to large-scale periodic treatment of affected populations with praziquantel, the only medicine recommended by the World Health Organization (WHO) against all forms of schistosomiasis. Regular treatment cures mild symptoms and prevents progression to severe, chronic disease. However, the burden of *S. mansoni* infection remains a public health problem since the risk of reinfection and recurrent disease exist even in areas with high treatment coverage in the country due to limited availability of praziquantel, and there is no suitable formulation of praziquantel for treatment of preschool aged children. Of note, schistosomiasis control with praziquantel has been successfully implemented over the past 40 years in several countries, including Brazil, Cambodia, China, Egypt, Mauritius, Islamic Republic of Iran, Oman, Jordan, Saudi Arabia, Morocco, and Tunisia.

With the aim of enhancing control programs of neglected tropical diseases across countries, WHO has issued a new road map plan for 2021–2030, which advocates enhanced financial and political support to achieve the target of interruption of transmission. Further plans, an integrated control strategy involving mass treatment with praziquantel, snail control and environmental modification, wider access to sanitation and safe water, economic and environmental development, health education and poverty-alleviation can greatly contribute to sustainable control and elimination of schistosomiasis. Additional research to address gaps, develop tools and optimize the impact of existing programs with snail control will complement the core strategic approach.

We observed a significant variation in the number of studies by region of Ethiopia with most studies conducted in Amhara (42%), Oromia (26%) and Southern Ethiopia (13%). The pooled prevalence of *S. mansoni* was 18% (95%CI: 14.0–23.0), which represents endemicity and moderate prevalence of *S. mansoni* infection nationally [111], which is lower than that reported in other settings, for example from Zambia (34.9%) [112]. The difference in prevalence may be due to differences in geographical and ecological variations, periodical cleaning of the irrigation canals, long time endemicity of study area, study design, sampling techniques, sample size, behavior of the study participants, environmental sanitation, and distribution of snails.

Southern Ethiopia has the highest prevalence (25.9%), followed by Tigray (20.2%) and Oromia (18.7%). The region has more water bodies than the other regions, which could be areas for harboring vectors that play a role in transmission. The variations across regions may also be explained by the differences in environmental conditions such as temperature and humidity, rainfall patterns and environmental sanitation which influence parasite transmission. Other factors may include availability and abundance of snail intermediate hosts, socioeconomic conditions, levels of community awareness of the disease, variations in study period, and methods of diagnosis, among others.

2Our study revealed a decrease in the prevalence of the disease during the last 15 years (2006–2020), probably due to the impact of control interventions by governmental and non-governmental bodies in Ethiopia [113]. However, the reduction was not significant despite control efforts potentially because of a recent water resources development for irrigation and intensive human migration in the country [114]. Furthermore, climate change and global warming which usually result in increased temperature may be additional factors [115]. For instance, a study from Nigeria showed that a rise in ambient temperature from 20 to 30 °C led to increase in the mean burden of *S. mansoni* infection [116].

Another important finding is the higher prevalence in rural settings which is similar to reports from Kinshasha, Congo [1, 117]. The higher prevalence from rural settings may be due to increased exposure to water through different activities such as irrigation practice, swimming and fishing, limited access to health-care services and lack of safe water for the rural population. Males were also more affected than females (28.5% versus 21.2%), which is similar to previous studies from Ethiopia [24, 118]. Men’s participation in outdoor activities like irrigation, farming and swimming and bathing in river water, could increase their risk of exposure to *S. mansoni* infection.

Higher pooled prevalence of *S. mansoni* was reported by Kato-Katz and formol-ether tests compared to other methods including wet mount, wet mount and formol-ether, which is possibly explained by the high sensitivity of Kato-Katz test for the diagnosis of *S. mansoni* infection. In the WHO 2002 report, higher sensitivity of kato-katz test in community with high infection intensity was shown [119]. It is important to note that these diagnostic tests are less sensitive/specific and tests like CCA/PCR would yield more realistic infection rates due to their sensitivity and specificity.

The prevalence of *S. mansoni* infection rate was high in children and adolescent than adolescent and adult or adults, which could be associated with children and adolescents are part takers in swimming and other activities increasing contact with water bodies. Similar results were reported in a review conducted in Nigeria [120].

### Limitation

Most studies included in the analysis were health facility-based studies, and the data might not be representative of the general population. Further, sample size variations, inconsistency of the laboratory diagnostic methods used in the studies as well as study time and regional heterogeneity may affect internal and external validity of our results. Another limitation of the review is that we excluded studies that used specific tests like CCA/PCR diagnostics.

## Conclusion and recommendation

The review showed a moderate prevalence of *S. mansoni* infection in Ethiopian and that the disease has remained to be a major health problem. Large-scale treatment of at-risk population groups, access to safe water, improved sanitation, hygiene education, and snail control would help to alleviate the burden of schistosomiasis. Given the national burden, there is a need to strengthen community engagement strategies through health education and develop prognostic models that can be used to show snail suitability areas to inform control measures. Further pooled data that showed factors contributing to endemicity of this parasite will be critical to develop effective control strategies.

## Supplementary Information

**Additional file 1.** The PRISMA Group (2009). Preferred Reporting Items for Systematic Reviews and Meta-Analyses.

**Additional file 2.** Study design and quality assessment of the studies included in systematic review and meta-analysis of S.mansoni in Ethiopia.

**Additional file 3.** List and characteristics of studies analyzed.

**Additional file 4.** Egger regression intercept for the prevalence of S.mansoni in Ethiopia.

**Additional file 5.** Meta regration for the prevalence of S.mansoni in Ethiopia.

## Data Availability

We do not want to share our data to use for another study.
