# Supplementary material for: Prevalence of Schistosoma mansoni infection in Ethiopia: a systematic review and meta-analysis
Source: Trop Dis Travel Med Vaccines. 2021 Feb 1;7:4. doi: 10.1186/s40794-020-00127-x (PMC7849146; doi:10.1186/s40794-020-00127-x)
Supplement: Supplementary file 2 — Additional file 2. Study design and quality assessment of the studies included in systematic review and meta-analysis of S.mansoni in Ethiopia. [file 40794_2020_127_MOESM2_ESM.docx]

Additional file 2: Study design and quality assessment of the studies included in systematic review and meta-analysis of *S.mansoni* in Ethiopia

| Author(s) ref | Q1 | Q2 | Q3 | Q4 | Q5 | Q6 | Q7 | Q8 | Q9 | Quality score (9%) |
| --- | --- | --- | --- | --- | --- | --- | --- | --- | --- | --- |
| Leykun J, [19] | Y | Y | Y | NR | NR | Y | NR | Y | NR | 5 |
| Leykun J, [20] | Y | Y | Y | Y | Y | NR | Y | Y | Y | 8 |
| Degu G et al.,[21] | Y | Y | NR | Y | Y | NR | NR | NR | NR | 5 |
| [NEGA](https://www.ajtmh.org/search?value1=NEGA+BERHE&option1=author&noRedirect=true) B et al. [22] | Y | Y | Y | Y | NR | Y | Y | Y | NR | 7 |
| [NegaB](https://www.ajtmh.org/search?value1=Nega+Berhe&option1=author&noRedirect=true)t al., [23] | Y | Y | Y | Y | NR | Y | Y | Y | NR | 7 |
| Alemu et al.,[24] | Y | Y | Y | Y | Y | Y | Y | Y | NR | 8 |
| Huruy K et al.,[25] | Y | Y | Y | Y | Y | Y | NR | Y | NR | 7 |
| Asrat A et al.,[26] | Y | Y | Y | Y | Y | Y | NR | Y | Y | 8 |
| Essa T et al.,[27] | Y | Y | Y | Y | Y | Y | Y | Y | NR | 8 |
| Abera B et al.,[28] | Y | Y | Y | Y | Y | Y | Y | Y | NR | 8 |
| Awoke W et al.,[29] | Y | Y | Y | Y | Y | Y | Y | Y | Y | 9 |
| Reta B&Erko B,[30] | Y | Y | Y | Y | Y | Y | NR | NR | NR | 6 |
| King JD et al.,[31] | Y | Y | Y | Y | Y | Y | NR | NR | NR | 6 |
| Fentie T et al.,[32] | Y | Y | Y | Y | Y | Y | NR | Y | NR | 6 |
| Abate A et.al.,[33] | Y | Y | Y | y | Y | Y | NR | NR | NR | 6 |
| Abay SM et.al.,[34] | Y | Y | Y | y | Y | Y | NR | NR | NR | 6 |
| Hailu T,[35] | Y | Y | Y | Y | Y | Y | Y | Y | NR | 8 |
| Alebie G et al.,[36] | Y | Y | Y | Y | Y | Y | Y | Y | NR | 8 |
| Aemero M et al.,[37] A | Y | Y | Y | Y | Y | Y | NR | Y | Y | 8 |
| Degarege A et al.,[38] | Y | Y | Y | Y | Y | Y | NR | Y | NR | 7 |
| Mathewos B et al.,[39] | Y | Y | Y | Y | Y | Y | Y | Y | NR | 8 |
| Mamo H et.al.,[40] | Y | Y | Y | Y | Y | Y | Y | Y | Y | 9 |
| GetnetA&Worku S [41] | Y | Y | Y | y | Y | Y | NR | NR | NR | 6 |
| BitewAet al.,[42] | Y | Y | Y | y | Y | Y | Y | NR | NR | 7 |
| Gashaw F et al.,[43] | Y | Y | Y | y | Y | Y | Y | Y | NR | 8 |
| Yetemwork A et al.,[44] | Y | Y | Y | y | Y | Y | NR | NR | NR | 6 |
| Getie S et al.,[45] | Y | Y | Y | y | Y | Y | NR | NR | NR | 6 |
| Degarege A et al.,[46] | Y | Y | Y | y | Y | Y | NR | NR | NR | 6 |
| Alemu, A et al.,[47] | Y | Y | Y | y | Y | Y | Y | Y | NR | 8 |
| Amor A et al.,[48] | Y | Y | Y | y | Y | Y | NR | Y | NR | 7 |
| Abdi M et.al.,[49] | Y | Y | Y | Y | Y | Y | Y | NR | NR | 7 |
| Feleke D et al.,[50] | Y | Y | Y | Y | Y | Y | NR | NR | NR | 6 |
| Shiferaw MB et al.,[51] | Y | Y | Y | Y | Y | Y | NR | NR | NR | 6 |
| Eshetu T et.al.,[52] | Y | Y | Y | Y | Y | Y | NR | NR | NR | 6 |
| AndargieA&Abera A,[53] | Y | Y | Y | Y | Y | Y | NR | Y | NR | 7 |
| NUTE A et al.,[54] | Y | Y | Y | Y | Y | NR | Y | Y | Y | 6 |
| Hailu T et al.,[55] | Y | Y | Y | Y | Y | Y | Y | Y | NR | 8 |
| Leta Get al.,[56] | Y | Y | Y | Y | Y | Y | NR | NR | NR | 6 |
| Gizaw Z et.al.,[57] | Y | NR | Y | Y | Y | Y | NR | Y | NR | 7 |
| Sitotaw B et.al.,[58] | Y | Y | Y | Y | Y | Y | Y | Y | Y | 9 |
| Kassaw M et.al.,[59] | Y | Y | NR | Y | Y | Y | NR | Y | Y | 7 |
| Workineh L et.al.,[60] | Y | Y | NR | Y | Y | Y | NR | Y | Y | 7 |
| Hailemariam G et al.,[61] | Y | Y | NR | NR | Y | Y | NR | Y | NR | 5 |
| Amare M et al.,[62] | Y | Y | Y | Y | Y | Y | Y | Y | Y | 9 |
| AbebeGe et al., [63] | Y | Y | Y | y | Y | Y | NR | NR | NR | 6 |
| MengistuM et. Al.,[64] | Y | Y | Y | Y | Y | Y | NR | Y | NR | 7 |
| Mekonnen Z et al.,[65] | Y | Y | NR | Y | Y | Y | NR | Y | Y | 7 |
| Dufera M et al.,[66] | Y | Y | Y | Y | Y | Y | Y | Y | NR | 8 |
| Yirgalem G/hiwot et al.,[67] | Y | Y | Y | Y | Y | Y | Y | NR | NR | 7 |
| Beyene and Tasew[68] | Y | Y | Y | Y | Y | Y | NR | Y | NR | 7 |
| Begna Tulu1,[69] | Y | Y | Y | Y | Y | Y | NR | NR | NR | 6 |
| Aemero M [37]O | Y | Y | Y | Y | Y | Y | NR | Y | Y | 8 |
| HailuT&Yimer M [70] | Y | Y | Y | Y | Y | Y | Y | Y | NR | 8 |
| Yimer M et al.,[71] | Y | Y | Y | Y | Y | Y | NR | Y | NR | 7 |
| Kure A et al., [72] | Y | Y | Y | y | Y | Y | NR | NR | NR | 6 |
| Jejaw A et al.,[73] | Y | Y | Y | Y | Y | Y | Y | Y | Y | 9 |
| Bajiro M etal.,[74] | Y | Y | Y | Y | Y | Y | Y | Y | NR | 8 |
| MekonnenZet al.,[75] | Y | Y | Y | Y | Y | Y | NR | NR | NR | 6 |
| Begna T et al., [76] | Y | Y | Y | Y | Y | Y | Y | Y | Y | 9 |
| Bajiro M et al.,[77] | Y | Y | Y | Y | Y | Y | NR | Y | NR | 7 |
| *Bajiro M* et al.,[78] | Y | Y | Y | y | Y | Y | NR | Y | NR | 7 |
| Teklemariam D et al.,[79] | Y | Y | Y | Y | Y | Y | NR | NR | NR | 6 |
| Mohammed Jet al.,[80] | Y | Y | Y | Y | Y | Y | NR | NR | NR | 6 |
| Kebede T et al.,[81] | Y | Y | Y | Y | Y | Y | NR | NR | NR | 6 |
| Bekana T et al.,[82] | Y | Y | Y | Y | Y | Y | Y | Y | Y | 9 |
| Sitotaw B et al., [ [83] | Y | Y | Y | Y | Y | Y | Y | Y | NR | 8 |
| Ansha M et al.,[84] | Y | Y | Y | Y | Y | Y | NR | Y | NR | 7 |
| Tefera et al.,[85] | Y | Y | Y | Y | Y | Y | NR | Y | NR | 7 |
| SamsonT et al.,[86] | Y | Y | Y | Y | Y | Y | NR | NR | NR | 6 |
| Assefa A et al.,[87] | Y | Y | Y | Y | Y | Y | NR | Y | NR | 7 |
| Mahmud MA, et al.,[88] | Y | Y | Y | Y | Y | Y | NR | Y | y | 8 |
| Desta H et al.,[89] | Y | Y | Y | Y | Y | Y | Y | Y | Y | 9 |
| Abebe N et al.,[90] | Y | Y | Y | Y | Y | Y | Y | NR | Y | 7 |
| Gebreegziabiher D et.al.,[91] | Y | Y | Y | NR | Y | Y | NR | NR | NR | 5 |
| Alemu M et al.,[92] | Y | Y | Y | Y | Y | Y | Y | Y | NR | 8 |
| Teshale T et al.,[93] | Y | Y | Y | Y | Y | Y | Y | Y | Y | 9 |
| GebreyohannsAet al.,[94] | Y | Y | Y | Y | Y | Y | Y | Y | NR | 8 |
| Nyantekyi LA et al.,[95] | Y | Y | Y | Y | Y | Y | NR | Y | NR | 7 |
| Ashenafi T etal.,[96] | Y | Y | Y | Y | Y | Y | NR | Y | NR | 7 |
| Erko B et al., [97] | Y | Y | Y | Y | Y | Y | Y | Y | Y | 9 |
| Wegayehu T et al., [98] | Y | Y | Y | Y | Y | Y | Y | NR | NR | 7 |
| Mulu A et.al.,[99] | Y | Y | Y | Y | Y | Y | NR | NR | NR | 6 |
| Aemero M et al.,[37] S | Y | Y | Y | Y | Y | Y | NR | Y | Y | 8 |
| Degarege A et al.,[100] | Y | Y | Y | Y | Y | Y | NR | Y | Y | 8 |
| AlemayehuB&Tomass Z [101] | Y | Y | Y | Y | Y | Y | Y | Y | NR | 8 |
| BerekeA et al.,[102] | Y | Y | Y | Y | Y | Y | Y | Y | Y | 9 |
| Tadege B &Shimelis T, [103] | Y | Y | Y | Y | Y | Y | Y | Y | Y | 9 |
| Grimes JE et.al.,[104] | Y | Y | Y | Y | Y | Y | Y | Y | NR | 8 |
| Tuasha N et.al., [ 105] | Y | Y | Y | Y | Y | Y | Y | Y | NR | 8 |
| Alemu G et.al.,[106] | Y | Y | Y | Y | Y | Y | Y | Y | NR | 8 |
| Girum T,[107] | Y | Y | Y | Y | Y | Y | Y | Y | NR | 8 |
| Teklemariam Zet al.,.[108] | Y | Y | Y | Y | Y | Y | NR | NR | NR | 6 |
| Negussu N et al.,[109] | Y | Y | Y | Y | Y | Y | NR | NR | NR | 6 |
| Kemal M et al.,[110] | Y | Y | Y | Y | Y | Y | Y | Y | NR | 8 |

**Key:** **Y**= Yes; **NR**= Not reported

**Question codes:**

1. Was the sample frame appropriate to address the target population?

2. Were study participants sampled in an appropriate way?

3. Was the sample size adequate?

4. Were the study subjects and the setting described in detail?

5. Wasthedataanalysisconductedwithsufficientcoverageoftheidentified sample?

6. Were valid methods used for the identification of the condition?

7. Was the condition measured in a standard, reliable way for all participants?

8. Was there appropriate statistical analysis?

9. Was the response rate adequate, and if not, was the low response rate managed appropriately?
