# Supplementary material for: Prevalence of Schistosoma mansoni infection in Ethiopia: a systematic review and meta-analysis
Source: Trop Dis Travel Med Vaccines. 2021 Feb 1;7:4. doi: 10.1186/s40794-020-00127-x (PMC7849146; doi:10.1186/s40794-020-00127-x)
Supplement: Supplementary file 3 — Additional file 3. List and characteristics of studies analyzed. [file 40794_2020_127_MOESM3_ESM.docx]

Additional file 3: List and characteristics of studies analyzed

| Authors | Pub. year | Region | Study  setting | Age group | Sample size | NO. pos. | Pre. (%) | Pre.(%)by Sex | | Diagnostic methods | Q. S |
| --- | --- | --- | --- | --- | --- | --- | --- | --- | --- | --- | --- |
|  |  |  |  |  |  |  |  | M | F |  |  |
| Leykun J, [19] | 1999 | Amhara | Rural & Urban | 5-20 yrs | 878 | 152 | 17.30 | NR | NR | Kato-Katz | 5 |
| Leykun J, [20] | 2001 | Amhara | Rural | 5-19 yrs | 687 | 133 | 19.4 | NR | NR | Kato-Katz | 6 |
| Degu G et al.,[21] | 2002 | Amhara | Urban | 10-14yrs | 165 | 84 | 50.8 | NR | NR | Kato-Katz | 5 |
| [NEGA](https://www.ajtmh.org/search?value1=NEGA+BERHE&option1=author&noRedirect=true) B et al.[22] | 2007 | Amhara | Rural | all | 421 | 309 | 73.4 | NR | NR | Kato-Katz | 7 |
| [NegaB](https://www.ajtmh.org/search?value1=Nega+Berhe&option1=author&noRedirect=true)t al., [23] | 2007 | Amhara | Rural | All age | 2451 | 1615 | 65.9 | NR | NR | Kato-Katz | 7 |
| Alemu et al.,[24] | 2011 | Amhara | Urban | 5 -17 yrs | 319 | 121 | 37.9 | 33.7 | 42.2 | Kato-Katz& wet mount | 8 |
| Huruy K et al.,[25] | 2011 | Amhara | Urban | all | 228 | 38 | 16.67 | 3.3 | 2.9 | Kato-Katz | 7 |
| Asrat A et al.,[26] | 2011 | Amhara | Rural & Urban | 7-25yrs | 704 | 112 | 15.9 | 51.6, | 48.4 | Wet mount & formal-ether | 8 |
| Essa T et al.,[27] | 2012 | Amhara | Rural & Urban | 7-24yrs | 579 | 119 | 20.6 | 18.4 | 22.7 | Wet mount & Kato-Katz | 8 |
| Abera B et al.,[28] | 2012 | Amhara | Rural | 7-14yrs | 778 | 57 | 7.3 | NR | NR | Kato-Katz,& formal-ether | 8 |
| Awoke W et al.,[29] | 2013 | Amhara | Rural & Urban | 5-24yrs | 828 | 7 | 0.8 | 0.6 | 0.2 | Formal-ether | 9 |
| Reta B&Erko B,[30] | 2013 | Amhara | Urban | 6-24yrs | 342 | 241 | 70.47 | 76.81 | 60.74 | Kato-Katz &formol-ether | 6 |
| King JD et al.,[31] | 2013 | Amhara | Rural | 2–15yrs | 2,338 | 68 | 2.9 | NR | NR | Kato-Katz &SAF | 6 |
| Fentie T et al.,[32] | 2013 | Amhara | Rural | 6-15yrs | 520 | 87 | 16.7 | NR | NR | Kato-Katz& formal-ether | 7 |
| Abate A et.al.,[33] | 2013 | Amhara | Urban | >1year | 410 | 36 | 8.9 | 62.1 | 62.3 | Wet mount &formol-ether | 6 |
| Abay SM et .al.,[34] | 2013 | Amhara | Urban | >5years | 152 | 28 | 18.4 | 7.1 | 2.5 | Kato-katz | 6 |
| Hailu T ,[35] | 2014 | Amhara | Urban | >1yrs | 2102 | 74 | 3.5 | NR | NR | Wet mount | 8 |
| Alebie G et al.,[36] | 2014 | Amhara | Rural | 5-19yrs | 384 | 318 | 82.8 | 83.9 | 81.7 | Kato-Katz & SAF | 8 |
| Aemero M et al.,[37] A | 2014 | Amhara | Rural & Urban | 5–60 yrs | 384 | 344 | 89.6 | 91 | 83.3 | Kato-Katz | 8 |
| Degarege A et al.,[38] | 2014 | Amhara | Urban | 8-12 yrs | 620 | 329 | 53.1 | NR | NR | Kato-Katz | 7 |
| Mathewos B et al.,[39] | 2014 | Amhara | Rural & Urban | 5-19 yrs | 261 | 88 | 33.7 | 28.7 | 39.3 | Kato-Katz | 8 |
| Mamo H et.al.,[40] | 2014 | Amhara | Urban | >15years | 236 | 0 | 0 | NR | NR | Wet mount &formol-ether | 9 |
| GetnetA&Worku S[41] | 2015 | Amhara | Rural & Urban | 7-18 yrs | 360 | 10 | 2.8 | 2.2 | 0.5 | Kato-Katz | 6 |
| BitewAet al.,[42] | 2015 | Amhara | Rural & Urban | All | 384 | 55 | 14.3 | NR | NR | Formal-ether | 7 |
| Gashaw F et al.,[43] | 2015 | Amhara | Urban | 5-17 | 550 | 270 | 49.0 | 54.5 | 44.1 | Kato-Katz | 8 |
| Yetemwork A et al.,[44] | 2015 | Amhara | Rural & Urban | 1-5 yrs | 277 | 5 | 1.9 | NR | NR | Wet mount &formol-ether | 6 |
| Getie S et al.,[45] | 2015 | Amhara | Rural & Urban | 5-19 yrs | 384 | 173 | 45.0 | NR | NR | Kato-Katz | 6 |
| Degarege A et al.,[46] | 2015 | Amhara | Rural | 1-80yrs | 484 | 129 | 26.65 | NR | NR | Kato-Katz | 6 |
| Alemu, A et al.,[47] | 2016 | Amhara | Rural | 6-6o yrs | 141 | 16 | 11.2 | 51.7 | 48.9 | Kato Katz | 8 |
| Amor A et al.,[48] | 2016 | Amhara | Rural | 5-18yrs | 396 | 62 | 15.7 | NR | NR | Formal-ether | 7 |
| Abdi M et.al.,[49] | 2016 | Amhara | Rural | 6-20years | 408 | 122 | 29.9 | 50.7 | 49.3 | Formal-ether | 7 |
| Feleke D et al.,[50] | 2017 | Amhara | Rural & Urban | 6 - 16 yrs | 279 | 213 | 76.5 | 28.4 | 15.5 | Wet mount &formol-ether | 8 |
| Shiferaw MB et al.,[51] | 2017 | Amhara | Rural & Urban | 15-49 yrs | 180 | 4 | 2.2 | NR | NR | Wet mount | 6 |
| Eshetu T et.al.,[52] | 2017 | Amhara | Urban | >20years | 223 | 2 | 0.9 | NR | NR | Wet mount | 6 |
| AndargieA&Abera A,[53] | 2018 | Amhara | Rural & Urban | >5 yrs | 228 | 38 | 16.67 | 76.5 | 23.5 | Kato-Katz and con | 7 |
| NUTE A et al.,[54] | 2018 | Amhara | Rural | 6-15yrs | 16,955 | 1170 | 6.9 | NR | NR | Wet mount &formol-ether | 8 |
| Hailu T et al.,[55] | 2018 | Amhara | Rural | 7-14 yrs | 409 | 33 | 8.0 | 21 | 7.6 | Ritchie’sl | 8 |
| Leta Get al.,[56] | 2018 | Amhara | Rural | 9-25yrs | 2650 | 355 | 13.4 | NR | NR | Kato-Katz | 6 |
| Gizaw Z et.al.,[57] | 2018 | Amhara | Rural | 6–59 months | 225 | 7 | 3 | NR | NR | Kato-Katz | 7 |
| Sitotaw B et.al.,[58] | 2019 | Amhara | Rural & Urban | 6-21yrs | 406 | 42 | 10.3 | 12 | 8.4 | Wet mount &formol-ether | 8 |
| Kassaw M et.al.,[59] | 2020 | Amhara | Rural & Urban | 2-6yrs | 378 | 141 | 37.2 | NR | NR | Wet mount &formol-ether | 8 |
| Workineh L et.al.,[60] | 2020 | Amhara | Rural | 5-18yrs | 340 | 6 | 1.8 | NR | NR | Kato-Katz | 7 |
| Hailemariam G et al.,[61] | 2004 | Oromia | Urban | >1yr | 78 | 2 | 2.5 | NR | NR | Wet mount &formol-ether | 5 |
| Amare M et al.,[62] | 2007 | Oromia | Urban | >1yrs | 754 | 11 | 1.5 | 22 | 14 | Wet mount &formol-ether | 9 |
| AbebeGe et al., [63] | 2009 | Oromia | Rural | NR | 198 | 21 | 10.61 | NR | NR | Wet mount &formol-ether | 6 |
| MengistuM et. Al.,[64] | 2011 | Oromia | Urban | >0years | 517 | 136 | 26.3 | 22.6 | 34.1 | Kato-Katz | 7 |
| Mekonnen Z et al.,[65] | 2014 | Oromia | Rural | 5-18 yrs | 453 | 241 | 53.2 | NR | NR | Kato-Katz | 7 |
| Dufera M et al.,[66] | 2014 | Oromia | Rural | >5yrs | 602 | 322 | 53.5 | 66.81 | 33.19 | Kato-Katz | 8 |
| Yirgalem G/hiwot et al.,[67] | 2014 | Oromia | Rural | <5yrs | 374 | 33 | 8.8 | 6.7 | 10.8 | Kato-Katz&SAF | 7 |
| Beyene and Tasew[68] | 2014 | Oromia | Urban | 1-15yrs | 260 | 1 | 0.4 | NR | NR | Wet mount &formol-ether | 7 |
| Begna Tulu1,[69] | 2014 | Oromia | Rural | 5-20yrs | 340 | 43 | 12.6 | 17.4 | 7.7 | formol-ether | 6 |
| Aemero M[37]O | 2014 | Oromia | Rural | 5–60 yrs | 345 | 207 | 59.9 | 65.5 | 55 | Formal-ether | 8 |
| HailuT&Yimer M [70] | 2014 | Oromia | Rural&Urban | 6-18yrs | 604 | 44 | 7.3 | 8.2 | 6.1 | Wet mount | 8 |
| Yimer M et al.,[71] | 2014 | Oromia | Rural | 1-20yrs | 422 | 239 | 56.6 | NR | NR | Kato-Katz | 7 |
| Kure A et al., [72] | 2015 | Oromia | Rural&Urban | 5-18yrs | 360 | 218 | 60.5 | NR | NR | Kato-Katz | 6 |
| Jejaw A et al.,[73] | 2015 | Oromia | Urban | 5 - 17 yrs | 460 | 206 | 44.8 | NR | NR | Wet mount &formol-ether | 9 |
| Bajiro M etal.,[74] | 2016 | Oromia | Rural | 6-18yrs | 500 | 120 | 24.0 | 25.6 | 22.5 | Kato-Katz | 8 |
| MekonnenZet al.,[75] | 2016 | Oromia | Rural | 1-90yrs | 1021 | 6 | 0.6 | NR | NR | Kato-Katz&wet.mo | 6 |
| Begna T et al., [76] | 2016 | Oromia | Rural | 6–18 yrs | 492 | 47 | 9.6 | NR | NR | Wet mount &formol-ether | 9 |
| Bajiro M et al.,[77] | 2017 | Oromia | Urban | 5-19yrs | 1000 | 84 | 8.40 | 13.4 | 3.4 | Kato-Katz | 7 |
| Bajiro M et al.,[78] | 2018 | Oromia | Urban | 5-19yrs | 233 | 62 | 26.6 | 29.2 | 23.3 | Kato Katz | 7 |
| Teklemariam D et al.,[79] | 2018 | Oromia | Rural | schoolchild | 280 | 100 | 35.7 | NR | NR | Kato-Katz &formol-ether | 6 |
| Mohammed Jet al.,[80] | 2018 | Oromia | Rural | >15yrs | 1011 | 361 | 35.7 | 31.5 | 30.1 | Kato-Katz | 6 |
| Kebede T et al.,[81] | 2018 | Oromia | Rural | 3-70yrs | 911 | 210 | 23.1 | 28.7 | 15.8 | Kato-Katz | 6 |
| Bekana T et al.,[82] | 2019 | Oromia | Urban | 6-15yrs | 317 | 136 | 42.9 | 51.2 | 33.1 | Kato-kataz & formal-ether | 9 |
| Sitotaw B et al.[ [83] | 2020 | Oromia | Rural & Urban | 5 to 15 years | 383 | 17 | 4.4 | 3.7 | 5.1 | Wet mount &formol-ether | 8 |
| Ansha M et al.,[84] | 2020 | Oromia | Urban | 5.9yrs | 298 | 34 | 11.4 |  |  | Kato-Katz | 7 |
| Tefera et al.,[85] | 2020 | Oromia | Urban | 7-17yrs | 328 | 94 | 28.7 | 39.3 | 12.9 | Kato-Katz | 7 |
| SamsonT et al.,[86] | 2000 | Tigray | Rural | <5yrs | 330 | 3 | 1.0 | NR | NR | Wet mount | 6 |
| Assefa A et al.,[87] | 2013 | Tigray | Urban | 6-19years | 457 | 109 | 23.9 | 30.71 | 14.12 | Kato-Katz | 7 |
| Mahmud MA,et al.,[88] | 2013 | Tigray | Rural&Urban | 6-15yrs | 600 | 84 | 14.0 | 18 | 10 | Kato-Katz and formal .con | 8 |
| Desta H et al.,[89] | 2014 | Tigray | Rural | 5-19yrs | 469 | 199 | 42.4 | 50.2 | 35.1 | Kato-Katz | 9 |
| Abebe N et al.,[90] | 2014 | Tigray | Rural | >1yrs | 371 | 274 | 73.9 | NR | NR | Kato-Katz | 6 |
| Gebreegziabiher D et.al.,[91] | 2014 | Tigray | Urban | 15-49 | 85 | 17 | 20 | NR | NR | Kato-Katz | 5 |
| Alemu M et al.,[92] | 2017 | Tigray | Rural & Urban | >15yrs | 427 | 14 | 3.3 | 1.9 | 4.7 | Wet mount & Kato-Katz | 8 |
| Teshale T et al.,[93] | 2018 | Tigray | Rural & Urban | 6-19yrs | 410 | 158 | 38.5 | 23 | 15.4 | Kato-Katz | 9 |
| GebreyohannsAet al.,[94] | 2018 | Tigray | Urban | 5-70yrs | 411 | 108 | 26.3 | 27.7 | 24.3 | Kato-Katz | 9 |
| Nyantekyi LA et al.,[95] | 2010 | Sothern | Rural | <5yrs | 288 | 107 | 37.20 | NR | NR | Kato-Katz and fomal.conc | 7 |
| Ashenafi T etal.,[96] | 2011 | Sothern | Rural | >5yrss | 419 | 309 | 73.7 | 76.6 | 70.6 | Kato-Katz technique | 7 |
| Erko B et al., [97] | 2012 | Sothern | Rural | 6-22yrs | 299 | 224 | 74.9 | 76.5 | 72.5 | Kato-Katz | 9 |
| Wegayehu T et al., [98] | 2013 | Sothern | Rural | all | 858 | 1 | 0.12 | 0.3 | 0 | Wet mount &formol-ether | 7 |
| Mulu A et.al.,[99] | 2013 | Sothern | Rural & Urban | >1year | 352 | 100 | 28.4 | NR | NR | Wet mount &formol-ether | 6 |
| Aemero M et al.,[37] S | 2014 | Sothern | Rural & Urban | 5–60 yrs | 344 | 109 | 31.6 | 32.8 | 30.2 | Kato-Katz thick smear | 8 |
| Degarege A et al.,[100] | 2014 | Sothern | Rural | 1-80yrs | 480 | 57 | 11.9 | 12.9 | 10.8 | Kato-Katz | 8 |
| AlemayehuB&Tomass Z [101] | 2015 | Sothern | Rural | 5- 16yrs | 384 | 312 | 81.3 | 85.1 | 77.1 | Kato-Katz&formol-ether | 8 |
| BerekeA et al.,[102] | 2017 | Sothern | Rural & Urban | 5-19yrs | 503 | 295 | 58.6 | 62.7 | 53.2 | Kato-Katz&formol-ether | 9 |
| Tadege B &Shimelis T, [103] | 2017 | Sothern | Rural | 5-15yrs | 374 | 116 | 31.0 | 31.9 | 29.5 | Formal-ether | 9 |
| Grimes JE et.al.,[104] | 2017 | Sothern | Rural | 5-18 years | 125 | 0 | 0.3 | NR | NR | Kato-kataz & formal-ether | 8 |
| Tuasha N et.al, [ 105] | 2019 | Sothern | Urban | 6-77yrs | 427 | 57 | 13.35 | NR | NR | Wet mount & Kato-Katz | 8 |
| Alemu G et.al.,[106] | 2019 | Sothern | Rural & Urban | 5–14 years | 351 | 4 | 1.1 | NR | NR | Wet mount &formol-ether | 8 |
| Girum T,[107] | 2005 | Harari | Urban | 5-24yrs | 422 | 18 | 4.3 | 6.3 | 0.7 | formol-ether | 8 |
| Teklemariam Zet al.,.[108] | 2013 | Harari | Rural & Urban | >15yrs | 259 | 2 | 0.8 | NR | NR | Wet mount &formol-ether | 6 |
| Negussu N et al.,[109] | 2013 | Afar | Rural | 5-17yrs | 523 | 0 | 0.0 | NR | NR | Kato-Katz | 6 |
| Kemal M et al.,[110] | 2019 | Somali | Rural & Urban | <5yrs | 236 | 59 | 25 | 23.72 | 16.94 | Kato-Katz | 8 |

NB: CS=Cross-sectional, NR=Not reported, formol-ether=Formaline ether concentration technique, SAF: Sodium acetate-acetic acid-formalin Yrs=Years, Pre.=prevalence NR=Not reported, Q_S=Quality score.
