# Supplementary material for: Prevalence of Schistosoma mansoni infection in Ethiopia: a systematic review and meta-analysis
Source: Trop Dis Travel Med Vaccines. 2021 Feb 1;7:4. doi: 10.1186/s40794-020-00127-x (PMC7849146; doi:10.1186/s40794-020-00127-x)
Supplement: Supplementary file 4 — Additional file 4. Egger regression intercept for the prevalence of S.mansoni in Ethiopia. [file 40794_2020_127_MOESM4_ESM.docx]

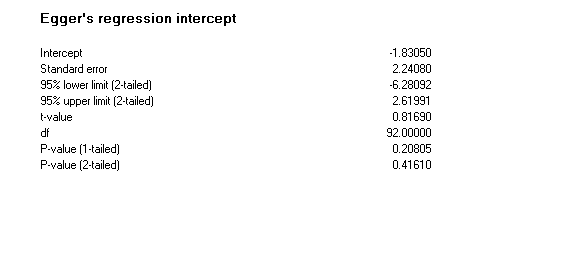


Additional file 4: Egger regression intercept for the prevalence of Schistosoma mansoni in Ethiopia
