# Supplementary figures and images for: Prevalence of Schistosoma mansoni infection in Ethiopia: a systematic review and meta-analysis
Source: Trop Dis Travel Med Vaccines. 2021 Feb 1;7:4. doi: 10.1186/s40794-020-00127-x (PMC7849146; doi:10.1186/s40794-020-00127-x)

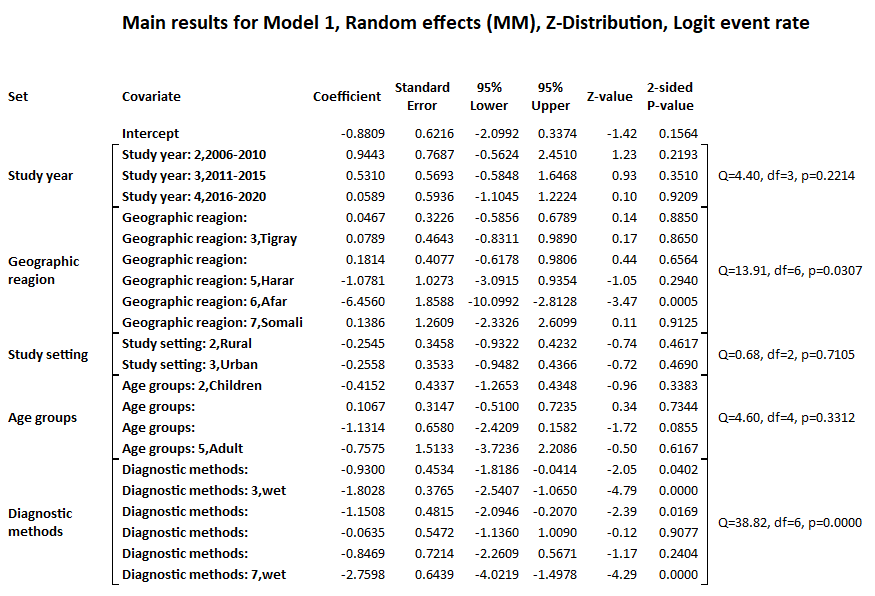


Additional file 5: Meta regration for the prevalence of Schistosoma mansoni in Ethiopia

Supplement: Supplementary file 5 — Additional file 5. Meta regration for the prevalence of S.mansoni in Ethiopia. [file 40794_2020_127_MOESM5_ESM.docx]
